# Administrative Progress Throughout 1908

**Published:** 1909-01-02

**Authors:** 


### NURSING ADMINISTRATION.

### ADMINISTRATIVE PROGRESS THROUGHOUT 1908.

THE controversy centring round the establishment of an official register or list of trained nurses has never reached so lively a stage as in the past year during the history of the movement. All that could possibly be said for the proposal has been said over and over again. The matter has been twice before the House of Lords, where it met with most courteous consideration, all the more genial, it may be divined, because the vote carried with it no sense of finality. It has been loudly maintained by its promoters that the measure is demanded by the whole body of nurses and merely obstructed by a few mischievous people who aim at subjecting an honourable profession of women. It is interesting, therefore, to study the list of adherents who have been gained over to the cause of registration after the wide advertisement bestowed on it by its conditional acceptance by the Lords and by the active propaganda carried on now for many years in its favour. There are 18 training schools for nurses in London, containing over 100 beds. Out of this number three matrons alone are in favour of the Bill. There are 59 provincial training schools in England containing over 100 beds. Out of this number 13 are in favour of the Bill. The figures speak for themselves, and are an eloquent comment on the statements made in the House of Lords and elsewhere. Among Poor-law matrons more adherents have been gained, 12 out of 22 in London having declared in favour of the Bill. But the conditions prevailing in rate- or State-supported institutions are unquestionably more favourable to the principles of registration and uniformity. The Poor-law Matrons' Association has done good work during the past year, and whatever changes may be coming with the reform of the Poor Law so anxiously awaited next year, it may be taken as certain that the matrons of these institutions will be found well prepared to take full advantage of them in the interests of the suffering poor. A notable increase of the staff in the case of the Wandsworth and the Portsmouth Poor-law infirmaries is a very encouraging feature in the history of rate-supported institutions during the past year.

The formation of the Fever Nurses' Association has been a step forward towards the rescue of these institutions from the imputation freely levelled against them in the past of being one of the main sources whence the pseudo-nurse gets launched into public employment. True, no very definite scheme has been so far evolved for putting a stop to the abuses connected with fever nursing, but the attention concentrated on the problems it presents by those intimately acquainted with the facts cannot fail to have good results in the future.

The Central Midwives Board has passed through a strenuous year, and may be now said to have got to grips with the complicated questions falling within its jurisdiction. The controversy with the Poor-law institutions, which at one time seemed to be passing into an acute stage, quieted down on the tardy publication of information defining the conditions on which it is thought advisable to "recognise" institutions as training schools for midwives. The training of midwives throughout England is still too greatly restricted, and ought, in the interests of the poor, to be far less concentrated, since the better-equipped training schools are almost entirely monopolised by women who expect to earn high fees among the well-to-do. The question of doctors' fees continues to be a burning one in many localities, and it is satisfactory to learn that the whole working of the Midwives Act is to be made a subject for inquiry by a departmental committee.

The work of the Jubilee Institute has made a great stride forward with the renewed appeal for its development. Another County Association, that for Worcestershire, has been added to its ranks, and it is to be regretted that the opposition of the "Holt Ockley" element in the Oxfordshire Federation bars the way to their affiliation with this great organisation.

The establishment of a preliminary training school in connection with the Bristol Royal Infirmary, on the model of the one at Guy's, marks an important advance in the spread of sound principles of education for pupil nurses. The success of this new departure will depend on the extent to which it can be made entirely self-supporting, for managers, we feel confident, only desire some security on this point before initiating such schools in connection with other large training schools.

In regard to originality, the honours of the year may be said to rest with the Victorian Nurses' Association at Melbourne for their fine scheme for conferring the matrons' diploma, successfully inaugurated this autumn by a course of lectures on Hospital Administration, followed by an examination in which 10 out of 20 candidates passed. The movement has in it the seeds of a long-needed reform, and may be hailed with all the more satisfaction because derived from Britain beyond the seas.

The Royal British Nurses' Association held their examination for nursing diplomas in April, when 29 candidates were successful and three failed, while at the examination in October the number of successful candidates was 18.

The record of those who have retired from active work during the year is a long one, and includes such well-known names as Miss Hepper, matron of St. Mark's Hospital, London; Miss Jane Husband, matron of the Maternity Hospital, Glasgow; Miss Sophie Morris, matron of the Bristol General Hospital; Miss Oxford, superintendent of Guy's Trained Nurses' Association; Miss McBride, theatre sister at Liverpool Royal Infirmary for 26 years; Miss M. E. Jones, matron of the General Hospital, Birmingham; Miss Oldacre, superintendent nurse at Aston Union Infirmary; Miss Gertrude Wyld, matron of the Holborn Infirmary at Upper Holloway; Miss Ogston, superintendent of the City Hospital for Infectious Diseases, Newcastle-on-Tyne; Miss Brewster, who devoted her services to the miners in West Cornwall.

